# NagC represses *N*-acetyl-glucosamine utilization genes in *Vibrio fischeri* within the light organ of *Euprymna scolopes*

**DOI:** 10.3389/fmicb.2015.00741

**Published:** 2015-07-17

**Authors:** Yan Sun, Subhash C. Verma, Haikel Bogale, Tim Miyashiro

**Affiliations:** Department of Biochemistry and Molecular Biology, The Pennsylvania State University, University Park, PA, USA

**Keywords:** *Vibrio*, symbiosis, NagC, *N*-acetyl-glucosamine, *Euprymna scolopes*

## Abstract

Bacteria often use transcription factors to regulate the expression of metabolic genes in accordance to available nutrients. NagC is a repressor conserved among γ-proteobacteria that regulates expression of enzymes involved in the metabolism of *N*-acetyl-glucosamine (GlcNAc). The polymeric form of GlcNAc, known as chitin, has been shown to play roles in chemotactic signaling and nutrition within the light organ symbiosis established between the marine bacterium *Vibrio fischeri* and the Hawaiian squid *Euprymna scolopes*. Here, we investigate the impact of NagC regulation on the physiology of *V. fischeri*. We find that NagC repression contributes to the fitness of *V. fischeri* in the absence of GlcNAc. In addition, the inability to de-repress expression of NagC-regulated genes reduces the fitness of *V. fischeri* in the presence of GlcNAc. We find that chemotaxis toward GlcNAc or chitobiose, a dimeric form of GlcNAc, is independent of NagC regulation. Finally, we show that NagC represses gene expression during the early stages of symbiosis. Our data suggest that the ability to regulate gene expression with NagC contributes to the overall fitness of *V. fischeri* in environments that vary in levels of GlcNAc. Furthermore, our finding that NagC represses gene expression within the squid light organ during an early stage of symbiosis supports the notion that the ability of the squid to provide a source of GlcNAc emerges later in host development.

## Introduction

Members of the bacterial family *Vibrionaceae* typically exhibit both free-living and host-associated lifestyles (Thompson et al., 2006). Such complex lifestyle transitions underscore the need to both efficiently utilize the nutrients available within a given environment and conserve energy by suppressing metabolic pathways for unavailable nutrients. Gene regulatory networks that link metabolite availability with transcription of associated metabolic enzymes represent a solution prevalent among microbes.

**FIGURE 1:**
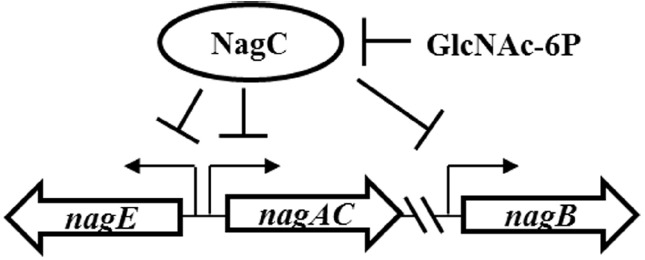
**Model of gene regulation by NagC in *V. fischeri*.** NagC represses the expression of genes, including *nagE*, *nagA*, and *nagB*. GlcNAc-6P, the intracellular form of GlcNAc, inhibits DNA binding by NagC through allosteric regulation.

*Vibrionaceae* members are able to degrade the polymeric form of *N*-acetyl-glucosamine (GlcNAc) known as chitin, and a conserved chitin-utilization pathway has been proposed for this bacterial family (Hunt et al., 2008). This pathway includes exochitinases, chitodextrinases, and β-*N*-acetylglucosaminidases, which break down chitin into GlcNAc and chitobiose ([GlcNAc]_2_). GlcNAc is predicted to be a preferred carbon source for members of the *Vibrionaceae* family (Hunt et al., 2008; Houot et al., 2010). Uptake of GlcNAc depends on the PTS transporter NagE, which phosphorylates the aminosugar to yield *N*-acetyl-glucosamine-6-phosphate (GlcNAc-6P) (Rogers et al., 1988). [GlcNAc]_2_ is predicted to cross the inner membrane through an ABC-type transporter and then be converted to GlcNAc-6P through the combined actions of *N,N′*-diacetylchitobiose phosphorylase, GlcNAc-1P-mutase and a GlcNAc-specific kinase (Hunt et al., 2008). *Vibrionaceae* genomes can possess at least two homologs of *nagA* (Hunt et al., 2008), which encodes a deacetylase that converts GlcNAc-6P to glucosamine-6P (GlcN-6P). Conversion of GlcN-6P to fructose-6-phosphate requires the deaminase NagB. The expression of genes encoding these GlcNAc-utilization genes is directly controlled by NagC (Figure 1), a transcriptional repressor conserved among γ-proteobacteria (Miyashiro et al., 2011). In response to the inducer GlcNAc-6P, NagC dissociates from promoter regions permitting the transcription of GlcNAc-utilization genes (Miyashiro et al., 2011).

*Vibrio fischeri* is a bioluminescent bacterium that forms a mutualistic symbiosis with the Hawaiian squid, *Euprymna scolopes* (Stabb and Visick, 2013). After hatching, juvenile squid acquire *V. fischeri* symbionts from the seawater environment. Within its light organ, *E. scolopes* houses populations of *V. fischeri* that emit bioluminescence that the host uses for an anti-predatory behavior known as counter-illumination (Jones and Nishiguchi, 2004). There is increasing evidence that *V. fischeri* is exposed to sources of GlcNAc derived from the host, particularly in the polymeric form of GlcNAc known as chitin. For example, to initially enter the pores of the light organ, *V. fischeri* cells use flagella-based motility and chemotaxis to swim up a gradient of chitin-derived oligosaccharides (COS; Mandel et al., 2012). Within deep crypt spaces of the light organ, *V. fischeri* also encounters chitin associated with macrophage-like haemocytes (Heath-Heckman and McFall-Ngai, 2011). As the squid host matures, a diel rhythm emerges that involves the provision of COS to *V. fischeri* symbionts (Wier et al., 2010; Schwartzman et al., 2015). By 4 weeks post-infection, *V. fischeri* Δ*nagB* mutants exhibit a persistence defect, presumably due to their inability to metabolize GlcN-6P generated during the metabolism of COS (Schwartzman et al., 2015).

NagC regulates several of the GlcNAc/chitin-utilization genes, including *nagB*, that vary in expression over the diel cycle (Wier et al., 2010; Miyashiro et al., 2011). NagC of *V. fischeri* was shown to function in similar fashion to other γ-proteobacteria: repression by NagC is relieved by the allosteric regulation mediated by GlcNAc-6P binding (Miyashiro et al., 2011). Previous work in *Escherichia coli* has demonstrated that NagC repression is important for cellular physiology in the absence of GlcNAc (Plumbridge, 1992). Here, we report our studies that characterize the impact of NagC regulation on *V. fischeri* physiology. In addition, we provide evidence of NagC repression within the light organ environment.

## Materials and Methods

### Strains and Plasmids

Strains and plasmids used in this study are listed in Table 1.

**Table 1:** **Strains and plasmids used in this study**.

	**Genotype**	**References**
**Strain**		
ES114	Wild-type *V. fischeri*	Mandel et al. (2008);
		Ruby et al. (2005)
TIM302	ES114 *att*Tn*7*::*gfp erm*	Miyashiro et al. (2010)
TIM381	ES114 Δ*nagC*::*nagC*E241A-*myc*-6x*his*	Miyashiro et al. (2011)
WPK100	ES114 Δ*nagC*	Miyashiro et al. (2011)
		
**Plasmid**		
pTM280	pVSV105 *P_tetA_-mCherry P_luxI_-gfp*	Miyashiro et al. (2010)
pTM355	pVSV105 *P_tetA_-mCherry P_nagA_-gfp*	Miyashiro et al. (2011)
pVSV105	R6Kori *ori*(pES213) RP4 *oriT cat*	Dunn et al. (2006)

### Growth Conditions and Media

*Vibrio fischeri* strains were grown aerobically at 28°C in LBS medium [1% (wt/vol) tryptone, 0.5% (wt/vol) yeast extract, 2% (wt/vol) NaCl, 50 mM Tris-HCl (pH 7.5)] or Tris-minimal medium [50 mM MgSO_4_, 10 mM CaCl_2_, 300 mM NaCl, 10 mM KCl, 0.0058% (wt/vol) K_2_HPO_4_, 10 μM FeSO_4_, 50 mM Tris-HCl (pH 7.5)] containing 0.4% glycerol and 0.1% ammonium acetate (TMM). For maintenance of plasmids in *V. fischeri*, 2.5 μg/ml chloramphenicol was used. TB-IO motility plates contained 0.5% tryptone, 0.3% yeast extract, and 0.25% agar in 70% Instant Ocean (Spectrum Brands, Blacksburg, VA, USA). Filter-sterilized seawater (FSSW) refers to 100% Instant Ocean filtered through a 0.2-μm surfactant-free filter.

### Gene Expression Measurements

For GlcNAc dose-response curves, the gene expression measurements were performed using fluorescence reporters as previously described (Miyashiro et al., 2014). Briefly, overnight LBS cultures of strains harboring reporter plasmids pTM355 [*nagA* (*VF_0807*)] or pTM280 [*luxI* (*VF_A0924*)] were diluted 1:100 into TMM containing the indicated level of GlcNAc and grown aerobically at 28°C with shaking. At OD_600_ ∼1.0, cultures were cooled quickly on ice, and 1-ml samples were spun at 15,000 × *g* for 2 min. The corresponding pellets were re-suspended in 350 μl cold TMM, and for each sample, three 100-μl technical replicates were measured with a Tecan M1000Pro fluorescence plate reader (Tecan Group, Mannedorf, Switzerland) for OD_600_, GFP (488 ± 5 nm excitation/509 ± 5 nm emission), and mCherry (587 ± 5 nm excitation/610 ± 5 nm emission) fluorescence. The non-fluorescent strain pVSV105/ES114 grown to OD_600_ ∼1.0 in parallel was used to account for auto-fluorescence.

For the gene expression measurements involving [GlcNAc]_2_, the experiments were performed as described above, except for the following steps. Cultures of 250-μl volume were grown in 5-ml Falcon tubes. At OD_600_ ∼1.0, cultures were concentrated by centrifugation at 15,000 × *g* for 2 min, and the pellets were re-suspended in 210 μl cold TMM. For each sample, two 100-μl technical replicates were measured as described above.

### Culture Competitions

For each competition involving TMM or LBS, overnight LBS cultures of TIM302 and a test strain were diluted 1:100 into the indicated growth medium and grown aerobically at 28°C. At OD_600_ ∼0.2–0.3, cultures were normalized to OD_600_ = 0.1, combined in a 2-ml volume, and grown at 28°C. At OD_600_ = 1.0, cultures were sampled for CFUs on LBS and diluted into fresh medium to OD_600_ ∼0.1. This sampling procedure was repeated two additional times. Generations were calculated as the number of doublings based on total CFU levels. The relative competitive index (RCI) was calculated as the ratio of non-fluorescent CFU to fluorescent CFU as normalized by the initial ratio. Fluorescence was scored using an Olympus SX16 fluorescence dissecting microscope (Olympus Corp., Tokyo, Japan) equipped with a GFP filter set.

For each competition involving FSSW, overnight LBS cultures of TIM302 and a test strain were diluted 1:100 into LBS and grown aerobically at 28°C. At OD_600_ ∼1.0, each culture was normalized to OD_600_ ∼0.05 and combined 1:1. For each competition, 4-μl of the mixture was diluted into 2 ml FSSW and incubated at room temperature. At the indicated time points, cultures were sampled for CFUs on LBS. The RCI was determined as described above.

### Motility Assays

Overnight LBS cultures of indicated strains were diluted 1:100 into LBS and grown aerobically at 28°C. At OD_600_ ∼0.2–0.3, a 2-μl sample was injected into a TB-IO motility plate and incubated at 28°C. After 4 h, a 5-μl sample of the indicated compound was spotted approximately 0.6 cm from the leading edge and incubated at 28°C. After 45 min, plates were scored for perturbation of the ring pattern.

### Light Organ Bacterial Gene Expression Measurements

Freshly-hatched juvenile *E. scolopes* squid were placed in FSSW containing approximately 10,000 CFU/ml of the indicated *V. fischeri* strains. At 16 h post-inoculation (p.i.), the squid were transferred to 4 ml FSSW. At 48 h p.i., animals were scored for luminescence and fixed overnight in 4% paraformaldehyde/1x marine phosphate buffer (mPBS) [50 mM Na phosphate (pH 7.4), 0.45 M NaCl]. Animals were washed several times in mPBS. Light organs were dissected, drained of ink, and mounted onto slides using VectaShield (Vector Laboratories Inc., Burlingame, CA, USA) as the mounting medium. DIC, GFP, and mCherry images were collected using a Zeiss 780 confocal microscope (Carl Zeiss AG, Jena, Germany) equipped with a 10x water lens. The confocal pinholes were set at maximum to create quantitative epi-fluorescence conditions. Optimal detector gain settings were determined for each promoter reporter.

To quantify gene expression levels, the mCherry image was converted to two binary mask images for bacterial populations and light organ using the max entropy and mean threshold algorithms of ImageJ 1.47v (NIH). Using the image toolbox of Matlab R2013a (MathWorks Inc., Natick, MA, USA), the host background signal was determined using the binary image constructed by using the non-zero pixels of the light organ mask image that did not overlap the non-zero pixels of the bacterial populations mask image. GFP and mCherry levels were determined by subtracting the corresponding average host background signals from the signals of the bacterial populations. Penn State does not require IACUC approval for invertebrate research.

## Results

### NagC Repression of *nagA* in Minimal Medium is Relieved by GlcNAc

To examine the impact of NagC on gene expression under controlled conditions, wild-type *V. fischeri* (ES114) was grown in TMM supplemented with various levels of GlcNAc. TMM also contained 0.4% glycerol and 0.1% ammonium acetate as carbon and nitrogen sources, which facilitated growth in low concentrations of GlcNAc. Transcriptional expression of *VF_0807*, which is the NagC-regulated gene *nagA* that encodes GlcNAc-6P deacetylase, was monitored using the promoter reporter plasmid pTM355 (Miyashiro et al., 2011). In this plasmid, the *nagA* promoter is located upstream of *gfp*, which encodes Green Fluorescent Protein (GFP). Also present in this plasmid is *mCherry*, which encodes the red fluorescent protein mCherry, under control of the *tetA* promoter, which is constitutively expressed in the absence of the TetR repressor.

**FIGURE 2:**
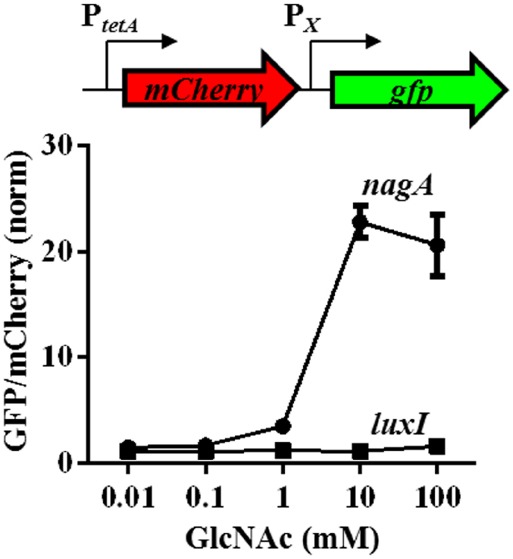
**Response of *nagA* expression to GlcNAc in *V. fischeri*.** Transcriptional response of various genes to GlcNAc in WT (ES114) harboring the reporter plasmids for *nagA* (pTM355) or *luxI* (pTM280) grown in TMM with the indicated level of GlcNAc. GFP/mCherry levels are normalized by WT cells grown in the absence of GlcNAc. Graphical points and error bars represent averages and SD of triplicate biological replicates, respectively. Experiment was performed three times, with similar results.

We found *nagA* transcription increased in a dose-dependent manner with exogenously added GlcNAc (Figure 2). Maximum induction of *nagA* expression occurred at 10 mM GlcNAc, with a level of expression 22-fold higher than that of un-induced cells (0 mM GlcNAc). In contrast, expression of *luxI*, which is not a member of the NagC regulon, remained constant over the range of GlcNAc used in the dose-response experiments.

### NagC Repression Improves Fitness of *V. fischeri* in the absence of GlcNAc

While conducting the *nagA* expression experiments described above, we observed that the cultures containing higher GlcNAc concentrations reached OD_600_ 1.0 prior to cultures containing lower levels of GlcNAc. In addition, we observed that a Δ*nagC* mutant grew more slowly than wild-type cells in TMM without supplemented GlcNAc (data not shown). From these observations, we hypothesized that NagC precisely tunes expression of the GlcNAc-utilization genes according to the availability of GlcNAc.

**FIGURE 3:**
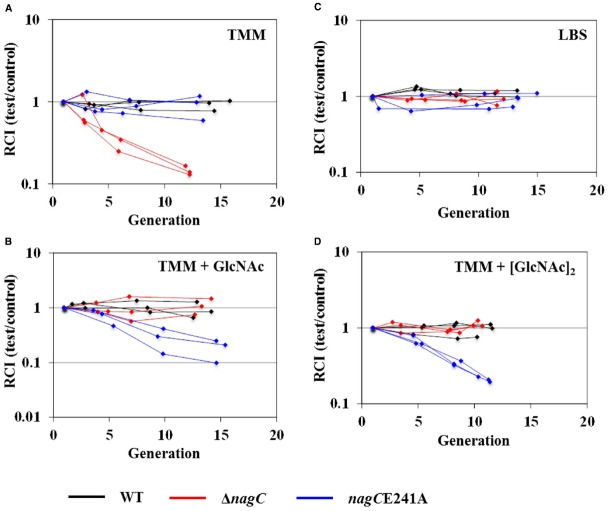
**Impact of NagC on fitness in minimal medium.** Relative competitive indices (RCI) of test strains WT (ES114; black), Δ*nagC* (WPK100; red), and *nagC*E241A (TIM381, blue) with control strain TIM302 (ES114 *gfp*). Each line represents an individual culture. Each experiment was performed twice, with similar results. (A) TMM. (B) TMM + 10 mM GlcNAc. (C) LBS. (D) TMM + 5 mM [GlcNAc]_2_.

To test this hypothesis, we compared the fitness of a Δ*nagC* mutant, which cannot regulate the GlcNAc-utilization genes, to that of wild-type cells grown in TMM ± 10 mM GlcNAc. As a measure of relative fitness, we used the RCI, which compares the CFU levels between test and control strains to the CFU ratio at the start of the experiment. For all of the competition assays performed in this study, the control strain was TIM302, which is a GFP-labeled, wild-type strain of *V. fischeri* (Miyashiro et al., 2010). In the absence of GlcNAc, the RCI of the Δ*nagC* mutant decreased, such that the relative abundance of the Δ*nagC* mutant was approximately 10-fold lower than TIM302 after 12 doublings (Figure 3A). However, no change in the RCI was detected for the Δ*nagC* mutant when the medium contained 10 mM GlcNAc (Figure 3B). Regardless of whether GlcNAc was present, the RCI of unlabeled, wild-type cells did not change over the course of the experiment. We also tested the Δ*nagC* mutant in LBS, which is a rich medium containing amino acids and peptides, and found that the RCI remained constant (Figure 3C). These results suggest that NagC repression contributes to the fitness of *V. fischeri* grown in defined medium in the absence of GlcNAc.

As another test of our hypothesis, we also conducted fitness experiments with a *V. fischeri* strain that expresses *nagC*E241A, which encodes a NagC variant that remains associated with DNA regardless of the presence of the GlcNAc-6P inducer (Miyashiro et al., 2011). The outcomes of the experiments with the *nagC*E241A mutant were opposite to those with Δ*nagC*: the *nagC*E241A mutant grew comparably to wild-type cells in the absence of GlcNAc (Figure 3A), but exhibited a decrease in RCI in the presence of 10 mM GlcNAc (Figure 3B). As observed with the Δ*nagC* mutant, the RCI of the *nagC*E241A mutant remained constant in LBS (Figure 3C). These results suggest that the ability of NagC to de-repress gene expression contributes to the fitness of *V. fischeri* in defined medium in the presence of GlcNAc.

### NagC De-repression Improves Fitness of *V. fischeri* in the Presence of [GlcNAc]_2_

**FIGURE 4:**
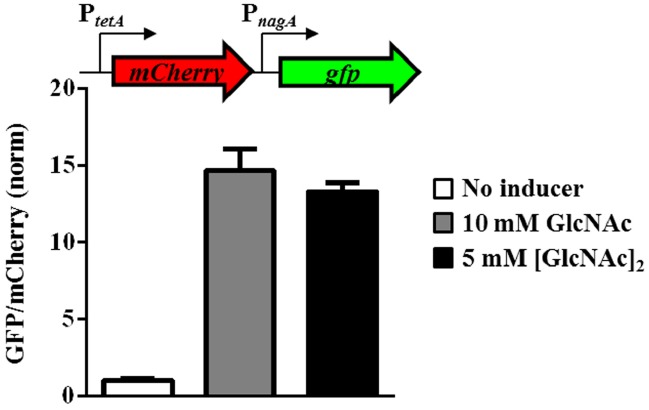
**Response of *nagA* expression to [GlcNAc]_2_ in *V. fischeri*.** Transcriptional response of *nagA* to GlcNAc and (GlcNAc)_2_ in WT (ES114) harboring the reporter plasmid for *nagA* (pTM355) grown in TMM. GFP/mCherry levels are normalized by WT cells grown without inducer. Graphical points and error bars represent averages and SD of triplicate biological replicates, respectively.

In *Vibrionaceae* members, the catabolism of COS is predicted to generate molecules of GlcNAc-6P (Hunt et al., 2008). To determine whether COS metabolism would lead to de-repression of NagC, we measured the expression of *nagA* in response to [GlcNAc]_2_, which is the smallest polymer of COS. We found that *nagA* expression was induced 13-fold in response to 5 mM [GlcNAc]_2_ (Figure 4). This response was comparable to that of *nagA* expression in response to 10 mM GlcNAc. This result suggests that NagC de-repression occurs in the presence of COS, such as [GlcNAc]_2_, presumably through the generation of GlcNAc-6P.

To address the impact of NagC regulation during metabolism of COS by *V. fischeri*, we adapted the competition assay by [GlcNAc]_2_. In the presence of 5 mM [GlcNAc]_2_, the Δ*nagC* mutant displayed wild-type growth, whereas the *nagC*E241A mutant exhibited a decrease in RCI (Figure 3D). These results, which are similar to those described above for GlcNAc, suggest that NagC de-repression is important for *V. fischeri* fitness when exposed to COS and are consistent with the proposed model for chitin utilization in *Vibrionaceae* (Hunt et al., 2008).

### NagC Repression Improves Fitness of *V. fischeri* in Seawater

**FIGURE 5:**
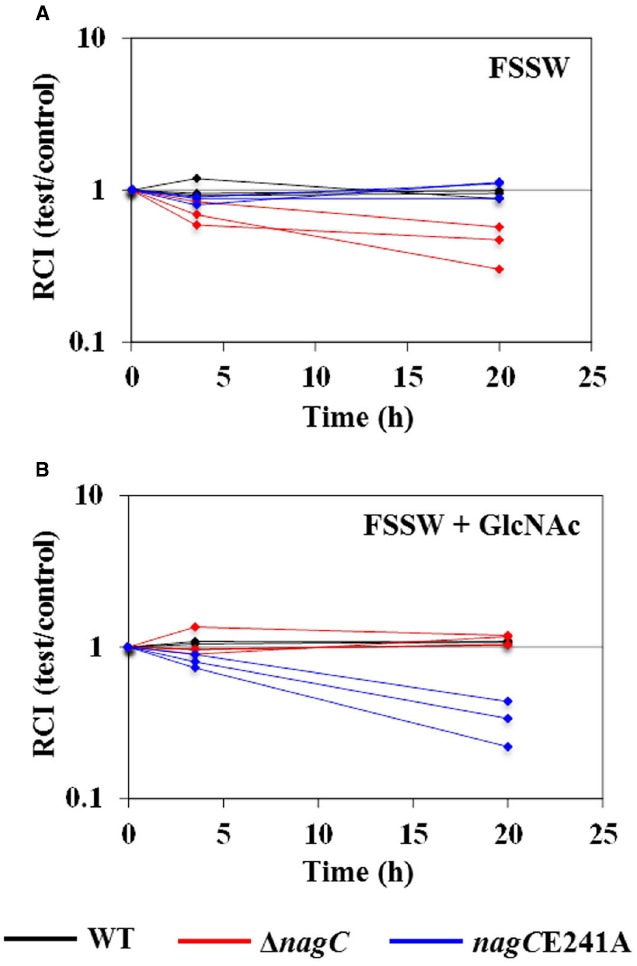
**Impact of NagC on fitness in filter-sterilized seawater.** RCI of test strains WT (ES114; black), Δ*nagC* (WPK100; red), and *nagC*E241A (TIM381, blue) with control strain TIM302 (ES114 *gfp*). Each line represents an individual culture. Each experiment was performed twice, with similar results. (A) FSSW. (B) FSSW + 10 mM GlcNAc.

NagC regulation was previously implicated as important for *V. fischeri* cells during the initial steps of colonization (Miyashiro et al., 2011). Our results described above suggest that the fitness of the Δ*nagC* cells is reduced in the absence of GlcNAc. Therefore, we investigated the fitness of Δ*nagC* in FSSW, which is the form of seawater used in our standard squid colonization assays. Similar to the results in TMM (Figure 3), we found that the RCI of the Δ*nagC* mutant decreased over time in FSSW (Figure 5A). This effect was abrogated in experiments that used FSSW supplemented with 10 mM GlcNAc (Figure 5B). In parallel, we examined the *nagC*E241A mutant and found that the RCI of *nagC*E241A decreased only in the presence of 10 mM GlcNAc (Figures 5A,B). Together, these data suggest that NagC repression is important for the fitness of *V. fischeri* in FSSW.

### Chemotaxis Toward GlcNAc is Independent of NagC Regulation in *V. fischeri*

Chemotaxis toward COS has been shown to promote colonization of the squid light organ by *V. fischeri* (Mandel et al., 2012). Our data suggest that both GlcNAc and COS (specifically [GlcNAc]_2_) lead to the de-repression of NagC. Therefore, we sought to test whether NagC regulation impacts the ability of *V. fischeri* to chemotax toward these compounds.

**FIGURE 6:**
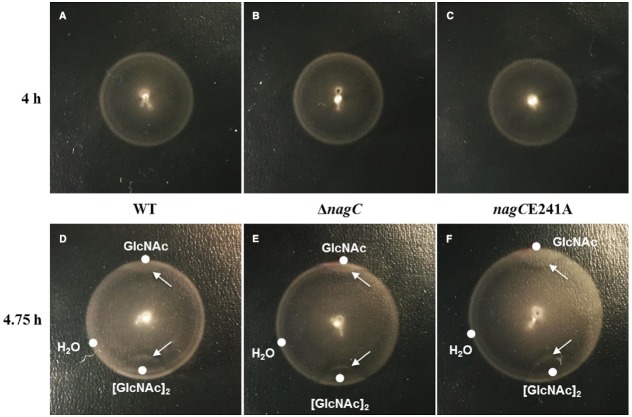
**Impact of NagC on chemotaxis toward GlcNAc and [GlcNAc]_2_.** Motility of WT (ES114); **(A,D)**, Δ*nagC* (WPK100); **(B,E)**, and *nagC*E241A (TIM381); **(C,F)** on TB-IO plates incubated at 28°C. Top panels show motility rings prior to placement of 5-μl volume of indicated compound. Bottom panels show migration 45 min after placement of compounds in the positions indicated by the dots. Arrows indicate the perturbation of the ring as a result of the supplemented compound.

We used Tryptone-Based Instant Ocean (TB-IO) soft-agar plates to evaluate the motility of *V. fischeri* strains containing mutations that impact NagC regulation. Both Δ*nagC* and *nagC*E241A mutants displayed wild-type motility, with the formation of two prominent rings by 4 h (Figures 6A–C). Within 45 min of spotting a 5-μl volume of either 20 mM GlcNAc or [GlcNAc]_2_, a gap had formed between the motility rings of wild-type cells indicating that the cells near the spot had migrated toward the source of GlcNAc or [GlcNAc]_2_ (Figure 6D). No response was observed for a spot containing water. This result is consistent with GlcNAc and [GlcNAc]_2_ being chemoattractants of *V. fischeri* (Cao et al., 2012; Mandel et al., 2012). The corresponding responses of Δ*nagC* and *nagC*E241A were indistinguishable relative to wild-type cells (Figures 6E,F). Together, these results suggest that NagC regulation does not impact the ability of cells to chemotax toward GlcNAc or [GlcNAc]_2_.

### NagC Represses Gene Expression Within Host-associated *V. fischeri* Populations

Previous work has demonstrated that while the incidence of light organ colonization by the Δ*nagC* mutant is reduced relative to wild-type cells, the mutant can achieve normal CFU levels once within the host (Miyashiro et al., 2011). In addition, a Δ*nagB* mutant, which cannot complete the metabolism of GlcNAc, is able to colonize the host at wild-type levels. Together, these results suggest that the levels of GlcNAc and COS within the juvenile light organ are insufficient to inhibit NagC repression.

**FIGURE 7:**
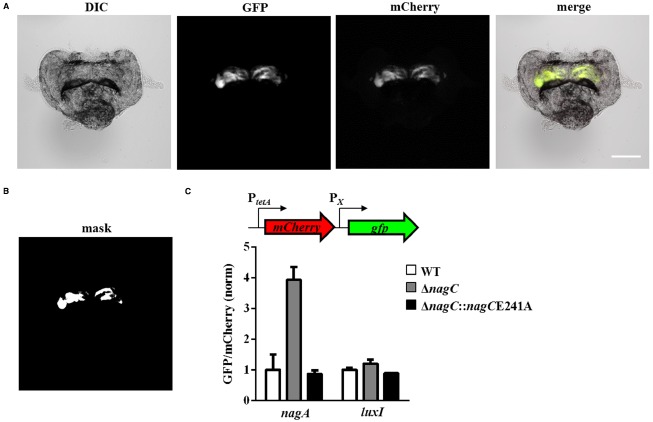
**NagC regulation of gene expression in squid light organ. (A)** (*L* to *R*) DIC, GFP, mCherry, and merged images of squid light organ colonized with *ΔnagC* harboring the *luxI* transcriptional reporter plasmid pTM280. Bar represents 200 μm. **(B)** Binary mask image generated by applying a thresholding algorithm to the mCherry image in **(A)**. The white pixels represent mCherry-expressing *V. fischeri* populations within the light organ shown in **(A)**. **(C)** Gene expression levels in squid colonized by WT (ES114), Δ*nagC* (WPK100), and Δ*nagC*::*nagC*E241A (TIM381) harboring transcriptional reporter plasmids for *nagA* (pTM355) and *luxI* (pTM280). For each promoter, the expression levels of each sample are normalized by wild-type levels. Graphical and error bars represent averages and SD of at least four light organs, respectively. Experiment was performed twice, with similar results.

To test this hypothesis, we developed an imaging method for quantifying gene expression within host-associated *V. fischeri* populations harboring the reporter plasmids shown in Figure 1. For each light organ, GFP and mCherry fluorescence levels were obtained by fluorescence microscopy (e.g., Figure 7A). The mCherry signal, which is constitutively expressed from the promoter reporters, was used to generate a binary mask image by a thresholding algorithm that indicates the position of *V. fischeri* populations within each image set (Figure 7B). Using this binary image, we quantified the corresponding levels of GFP and mCherry and calculated the GFP/mCherry fluorescence ratio. We found that the expression level of *nagA* in the Δ*nagC* mutant was 3.9-fold higher than the wild-type strain ES114 (Figure 7C). Furthermore, *nagA* expression in the *nagC*E241A mutant was comparable to the wild-type level. In contrast, the expression of *luxI* was similar among the strains used in these studies. Together, these results suggest that NagC represses *nagA* expression within *V. fischeri* populations inside the squid light organ.

## Discussion

In this study, we have found that NagC regulation impacts the fitness of *V. fischeri* in environments containing GlcNAc or COS. In particular, Δ*nagC* cells, which cannot repress GlcNAc-utilization genes, have decreased fitness in the absence of GlcNAc (Figure 3). Cells that are unable to de-repress GlcNAc-utilization genes also show decreased fitness in the presence of GlcNAc or COS, e.g., [GlcNAc]_2_, presumably through generation of GlcNAc-6P. In addition, the reduced fitness observed for the *nagC*E241A mutant grown in either GlcNAc or [GlcNAc]_2_ is consistent with the notion that GlcNAc-6P is a breakdown product during [GlcNAc]_2_ metabolism. We obtained similar results when cells were introduced into the seawater that is typically used to initiate squid colonization assays (Figure 5). We also found that chemotaxis toward GlcNAc and [GlcNAc]_2_ is independent of NagC regulation (Figure 6). Within the squid light organ, our data show that NagC actively represses gene expression (Figure 7).

**FIGURE 8:**
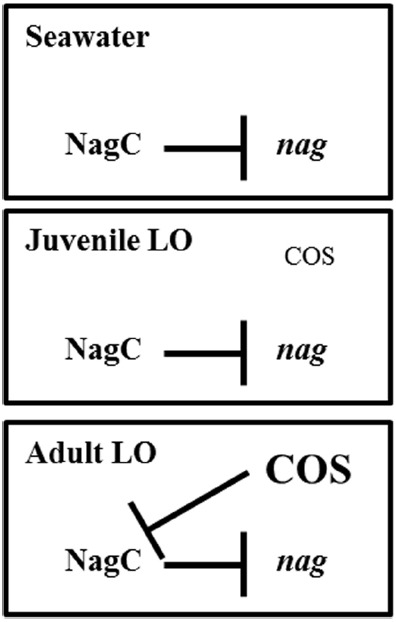
**Model for NagC function in symbiosis.** In nutrient poor conditions, including seawater, NagC represses gene expression. During initial colonization of the squid light organ, NagC continues to repress gene expression, despite the presence of a COS gradient that facilitates chemotaxis into the light organ pores (Mandel et al., 2012). Within the mature light organ, populations of *V. fischeri* use NagC to regulate gene expression in accordance with a diel rhythm involving COS (Wier et al., 2010; Schwartzman et al., 2015).

From these results, we present the model in Figure 8. In seawater, which contains less than 100 nM free monosaccharide sugars (Mopper et al., 1992), NagC represses the *nag* genes. During the initial colonization of the squid light organ, *V. fischeri* cells near the pores are exposed to a gradient of COS (Mandel et al., 2012). The concentration of COS within the squid light organ serves as a chemoattractant but is insufficient to de-repress gene regulation by NagC. Squid colonization assays involving Δ*nagC* alone (Miyashiro et al., 2011) or a transposon library (Brooks et al., 2014) both suggest that NagC repression during the initial stages of colonization is important. However, as the light organ matures, the levels of COS, and potentially GlcNAc, generated over the diel cycle become sufficient for de-repression of GlcNAc-utilization genes. In particular, expression of *VF_1598*, *VF_2139*, *VF_A0143*, and *VF_A0715*, which are predicted to be involved in chitin utilization and regulated by NagC (Miyashiro et al., 2011), increases at night, when COS is provided to the *V. fischeri* populations, and decreases at dawn (Wier et al., 2010; Schwartzman et al., 2015). Thus, we predict that NagC repression cycles on and off as *V. fischeri* experiences GlcNAc/COS levels that fluctuate over the diel cycle within the squid light organ.

Our experiments using LBS, a rich medium with free amino acids and peptides, have revealed that growth of *V. fischeri* is independent of NagC regulation (Figure 3C). While the NagC repression contributes to the fitness of *V. fischeri* in FSSW, once in the squid, accommodation of *V. fischeri* became independent of NagC regulation (Miyashiro et al., 2011). This finding supports the previous claim that growth of *V. fischeri* cells within the juvenile light organ primarily depends on their acquisition of host-derived free amino acids and peptides (Graf and Ruby, 1998).

The emergence of the diel rhythm associated with COS provision occurs between 2 and 4 weeks p.i., as evident by attenuated levels of a Δ*nagB* mutant at 4 weeks p.i. but not at 2 weeks p.i. (Schwartzman et al., 2015). The time period during which NagB function is important for the symbiosis occurs well after the host has selected for *V. fischeri* from the environment (∼4 h p.i.) and for luminous strains (∼2–5 days p.i.) (Koch et al., 2014). This observation suggests that NagC-regulated factors contribute neither to host-microbe specificity nor to the initial selection for luminescence function. Instead, our model suggests that the diel rhythm within the mature squid-vibrio symbiosis capitalizes on a core regulatory network common to γ-proteobacteria. As a consequence, we speculate that GlcNAc/COS-utilization genes used within the mature symbiosis are not subject to strong selection from host-microbe co-evolution.

This model involving a core metabolic regulatory network appears to differ from analogous pathways involved in gut microbial consortia. A recent study of homologous glycan-utilization genes in different *Bacteroides* species discovered that their transcriptional responses to the mammalian glycan chondroitin sulfate are specific to each species (Raghavan and Groisman, 2015). Furthermore, the response patterns did not correlate with species relatedness, which supports the conclusion that niche specificity within the mammalian gut selects for gene regulatory network architectures appropriate for the corresponding glycan availability, thereby contributing to *Bacteroides* diversity. The complexity associated with the gut microbial community is in stark contrast to the binary nature of the squid-vibrio symbiosis.

In summary, our studies highlight how NagC regulation contributes to the fitness of *V. fischeri* in environments that involve GlcNAc or COS. We anticipate that NagC regulation will vary as the light organ symbiosis matures and exhibits a diel rhythm. Recent efforts to raise the juvenile squid to adulthood (Koch et al., 2014; Schwartzman et al., 2015) suggest experiments designed to study NagC regulation in mature animals will be feasible. This work, which demonstrates that NagC functions as a repressor within the juvenile light organ, provides a foundation for such future directions.

### Conflict of Interest Statement

The authors declare that the research was conducted in the absence of any commercial or financial relationships that could be construed as a potential conflict of interest.
